# Single‐cell analysis reveals transcriptomic reprogramming in aging primate entorhinal cortex and the relevance with Alzheimer's disease

**DOI:** 10.1111/acel.13723

**Published:** 2022-09-27

**Authors:** Ming‐Li Li, Shi‐Hao Wu, Bo Song, Jing Yang, Li‐Yuan Fan, Yang Yang, Yun‐Chao Wang, Jing‐Hua Yang, Yuming Xu

**Affiliations:** ^1^ Department of Neurology The First Affiliated Hospital of Zhengzhou University, Zhengzhou University Zhengzhou Henan China; ^2^ School of Medicine Yunnan University Kunming Yunnan China; ^3^ Clinical Systems Biology Laboratories, Translation Medicine Center The First Affiliated Hospital of Zhengzhou University, Zhengzhou University Zhengzhou Henan China

**Keywords:** aging, alzheimer’s disease, entorhinal cortex, primate, single cell

## Abstract

The entorhinal cortex is of great importance in cognition and memory, its dysfunction causes a variety of neurological diseases, particularly Alzheimer's disease (AD). Yet so far, research on entorhinal cortex is still limited. Here, we provided the first single‐nucleus transcriptomic map of primate entorhinal cortex aging. Our result revealed that synapse signaling, neurogenesis, cellular homeostasis, and inflammation‐related genes and pathways changed in a cell‐type‐specific manner with age. Moreover, among the 7 identified cell types, we highlighted the neuronal lineage that was most affected by aging. By integrating multiple datasets, we found entorhinal cortex aging was closely related to multiple neurodegenerative diseases, particularly for AD. The expression levels of *APP* and *MAPT*, which generate β‐amyloid (Aβ) and neurofibrillary tangles, respectively, were increased in most aged entorhinal cortex cell types. In addition, we found that neuronal lineage in the aged entorhinal cortex is more prone to AD and identified a subpopulation of excitatory neurons that are most highly associated with AD. Altogether, this study provides a comprehensive cellular and molecular atlas of the primate entorhinal cortex at single‐cell resolution and provides new insights into potential therapeutic targets against age‐related neurodegenerative diseases.

## Graphical abstract

We provided the first single‐nucleus transcriptomic map of primate entorhinal cortex aging. Our result revealed that synapse signaling, neurogenesis, cellular homeostasis, and inflammation‐related genes and pathways changed in a cell‐type‐specific manner with age. By integrating multiple datasets, we found entorhinal cortex aging was closely related to multiple neurodegenerative diseases, particularly for Alzheimer's disease.

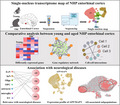

## 1 INTRODUCTION

Improvements in public health and medical treatment have greatly contributed to longer human life spans. However, susceptibility to a host of diseases, including diabetes (Kalyani et al., 2017), stroke (Yousufuddin & Young, 2019), cancer (Aunan et al., 2017), and neurodegeneration (Hou et al., 2019), increases with age. How to achieve healthy aging and delay functional degeneration has become an important issue.

The entorhinal cortex is situated in the medial temporal lobe, below the cerebral cortex near the hippocampus (Garcia & Buffalo, 2020). It forms circuits with different brain regions (Schultz et al., 2015), such as the hippocampus, amygdaloid nucleus, and neocortex (Gerlei et al., 2021). It processes information generated by the cerebral cortex and sends it to the hippocampus and amygdala, and vice versa. Thus, the entorhinal cortex is the “interface” for continuous information exchange between the hippocampus and neocortex (Sirota et al., 2003), and plays a crucial role in the acquisition, retrieval, and extinction of many forms of learning and memory (Coutureau & Di Scala, 2009; Eichenbaum et al., 2007). Pathological changes in the entorhinal cortex are associated with a variety of neurological diseases, particularly Alzheimer's disease (AD) (Khan et al., 2014). The entorhinal cortex is one of the first cortical brain regions to exhibit neuronal loss in AD (Braak & Braak, 1995; Leng et al., 2021).In addition, entorhinal cortex is among the first cortical fields to accumulate formation of β‐amyloid (Aβ) and neurofibrillary tangles (NFTs) in AD brains (Huijbers et al., 2014; Knopman et al., 2019). Therefore, a comprehensive understanding of the mechanisms underlying aging in the entorhinal cortex could provide insight into disease mechanisms and lead to therapeutic strategies.

Non‐human primates (NHPs), such as cynomolgus monkeys, are similar to humans in terms of entorhinal cortex structure, anatomical location, and function (Garcia & Buffalo, 2020), Therefore, analysis of the entorhinal cortex isolated from monkeys will help to better understand the etiology of aging‐related memory loss and cognitive decline(M.L. Li et al., 2019). Given the cellular heterogeneity of the entorhinal cortex(Kim & Park, 2021), the application of single‐cell/nucleus RNA sequencing (scRNA‐seq/snRNA‐seq) could expand our understanding of how cell types are affected during entorhinal cortex aging (J. Li et al., 2021; H. Zhang et al., 2021; W. Zhang et al., 2020).

Here, we obtained a single‐nuclear transcriptome atlas of the monkey entorhinal cortex as well as clarified gene and pathway alterations in a cell‐type‐specific manner during entorhinal cortex aging. Moreover, we integrated multiple neurodegenerative disease datasets based on single‐cell transcriptome data to clarify the correlation between disease and entorhinal cortex aging. This study advances our understanding of entorhinal cortex aging at the single‐cell level and elucidates potential therapeutic targets for interventions against neurodegenerative diseases in humans.

## 2 RESULTS

### 2.1 Single‐nucleus transcriptome map of NHP entorhinal cortex

**FIGURE 1:**
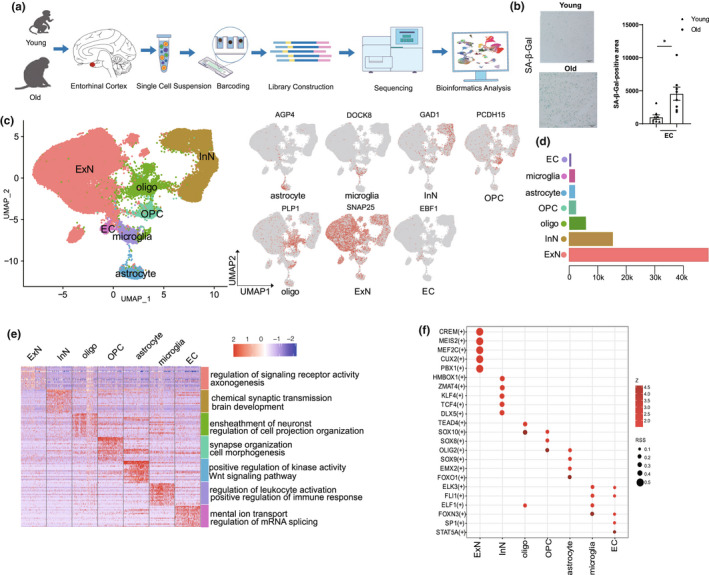
Construction of single‐nucleus atlas of entorhinal cortex by snRNA‐seq. (a). Study flowchart. (b). Immunofluorescence staining of SA‐β‐gal in entorhinal cortex of young and old monkeys. Scale bars, 50 μm.* *p* < 0.05. (c) Single‐nucleus transcriptional atlas of NHP entorhinal cortex. Uniform manifold approximation and projection (UMAP) plots showing different cell types by snRNA‐seq. (d). Number of each cell type in entorhinal cortex. (e). Heatmap showing expression profiles of indicated cell‐type‐specific marker genes of corresponding cell types in NHP entorhinal cortex. (f). Dot plot showing cell‐specific transcriptome regulons. Size of dot represents specific degree of TF; color of dot represents target gene number of TF

We collected the entorhinal cortex from young (7–8 years old) and aged (16–18 years old) cynomolgus monkeys (*Macaca fascicularis*) (Figure 1a; Table S1). The aged entorhinal cortices were characterized by higher senescence‐associated β‐galactosidase (SA‐β‐Gal) staining (Figure 1b), a common feature of senescent cells (Rodrigue et al., 2012). In addition, the accumulation of amyloid‐β (Aβ) deposits (immunostained by pan‐specific anti‐Aβ (4G8)) were significantly increased in aged entorhinal cortex (Figure S1 a) and overall neuronal density was significantly decreased in the aged entorhinal cortex (Figure S1 b).

To analyze cell populations and molecular characteristics, we performed snRNA‐seq on the entorhinal cortex of the cynomolgus monkeys (Figure 1a). After cell quality control and filtering, 76,839 single cells were retained for downstream analyses. Using unbiased clustering and uniform manifold approximation and projection (UMAP) analysis, we identified seven cell types in the entorhinal cortex based on classic cell‐type‐specific markers (Figure 1c; Table S2), including excitatory neurons (ExN, 48,687), inhibitory neurons (InN, 15,178), oligodendrocytes (5859), oligodendrocyte precursor cells (OPCs, 2283), astrocytes (2066), microglia (2047), and endothelial cells (ECs, 719) (Figure 1d). Gene Ontology (GO) enrichment analysis of cell‐type‐specific marker genes revealed the characteristics of each cell type. For example, the axonogenesis pathway was enriched in ExN genes, chemical synapse transmission pathway was enriched in InN genes, neuron projection development pathway was enriched in oligodendrocyte genes, and inflammatory response‐related pathway was enriched in microglial genes (Figure 1e). These results revealed the cellular heterogeneity in entorhinal cortex.

Furthermore, we identified the upstream regulators that drive cell differentiation in the entorhinal cortex. For example, the regulons of *CREM* and *MEIS2*, which are involved in cell differentiation and neurodegeneration (Mantamadiotis et al., 2002), were crucial regulators for neuronal lineage differentiation (ExN/InN) (Figure 1f, Table S3). The regulons of *FLI1*, which regulate inflammation‐associated genes (B. Chen et al., 2022), were identified as upstream regulators of microglia (Figure 1f, Table S3). We also identified several transcription factors (TFs), including *TEAD4*, *SOX10*, *SOX8*, and *OLIG2*, that regulate oligodendrocyte lineage differentiation (Figure 1f, Table S3). Together, our results clarify the cellular characteristic in the entorhinal cortex, providing the first single‐nucleus transcriptomic map of the entorhinal cortex in NHPs.

### 2.2 Neuronal lineage is most affected by entorhinal cortex aging

**FIGURE 2:**
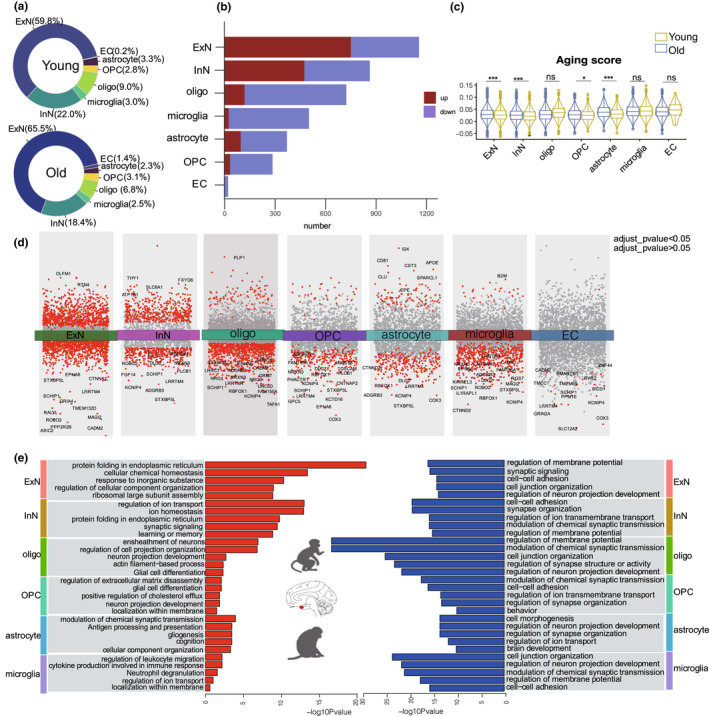
Age‐related transcriptional changes in various cell types of entorhinal cortex. (a). Pie chart showing percentage of cell population in different groups. (b). Bar plot showing number of DEGs across different cell types in NHP entorhinal cortex. (c). Violin plot showing gene set score of aging‐related genes across cell types. (d). Differential gene expression analysis showing up‐ and down‐regulated genes across all seven clusters. An adjusted *p* value <0.05 is indicated in red, while an adjusted *p* value ≥0.05 is indicated in gray. (e). Bar plot showing GO terms enriched in aging‐related DEGs of different cell types in entorhinal cortex. Red bar on left represents biological processes of up‐regulated genes in aged entorhinal cortex; blue bar on right represents biological processes of down‐regulated genes in aged entorhinal cortex

We next examined cell type‐specific transcriptional changes in the entorhinal cortex during aging. Comparing the relative cell proportions between young and aged NHP entorhinal cortices by multivariate test (Smillie et al., 2019), we found no significant changes in any cell types (Figure 2a; Figure S2). Next, we analyzed differentially expressed genes (DEGs) between young and aged entorhinal cortices according to cell type. The highest number of DEGs was observed in the neuronal lineage (Figure 2b). Moreover, by assessing the gene set scores of aging‐related genes across cell types in the entorhinal cortex (Aging Atlas, 2021), we found aging‐related genes were activated in multiple cell types derived from the aged entorhinal cortex, particularly neurons (Figure 2c). Together, our results suggest that neurons are most affected by entorhinal cortex aging.

### 2.3 Transcriptomic reprogramming in aging primate entorhinal cortex

DEGs analysis revealed significant changes in several key genes during entorhinal cortex aging (Figure 2d, Table S4). For instance, *OLFM*, which regulates neural progenitor maintenance and axon growth (Nakaya et al., 2012), was the most significantly up‐regulated gene in the ExNs, suggesting abnormal neurogenesis in the aged entorhinal cortex. *APOE*, which plays a role in lipid metabolism, Aβ aggregation, and tau damage (Yin & Wang, 2018), was up‐regulated in the astrocytes. *B2M*, which is a component of the MHC‐I molecule and accumulates during inflammation (Batista Muñoz et al., 2019), was up‐regulated in the microglia, thus suggesting elevated inflammation in the aged entorhinal cortex. These dysregulated genes may underlie the progressive functional decay of entorhinal cortex cells during aging.

GO enrichment analysis of DEGs revealed the cellular pathways involved in entorhinal cortex aging (Figure 2e). The synapse signaling pathway was down‐regulated in all cell types, while pathways associated with up‐regulated genes exhibited diversity across cell types. For example, the ExN‐up‐regulated genes were primarily involved in protein folding in the endoplasmic reticulum and cellular chemical homeostasis, suggesting dysregulation of homeostasis in aged ExNs (Estébanez et al., 2018). Neuronal projection organization was up‐regulated in the oligodendrocytes and OPCs, and inflammation‐related pathways were up‐regulated in the microglia. Together, our result clarified the profile of transcriptomic reprogramming in aging primate entorhinal cortex.

**FIGURE 3:**
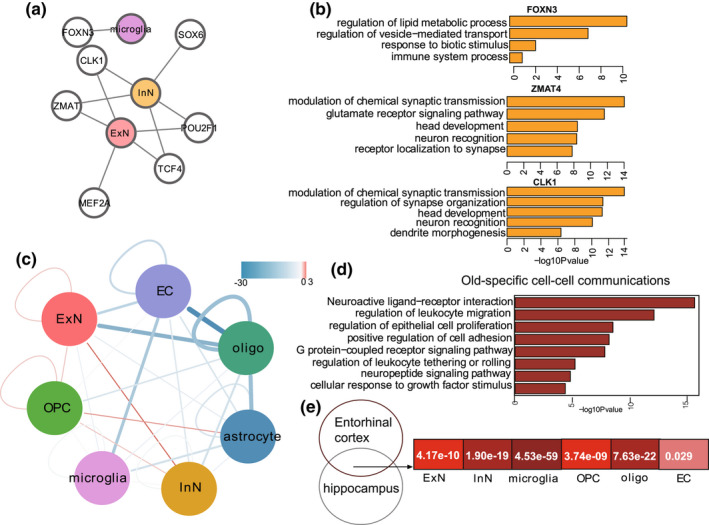
(a). Upstream regulons that drive cell‐type‐specific state transitions toward aging (b). GO enrichment analysis indicated that downstream DEGs targeted by upstream regulons. (c). Network plot showing cell–cell communication changes between cell types in entorhinal cortex. Color of connecting lines indicates number of altered interaction pairs. Red, increased interactions; blue, decreased interactions. (d). Bar plot showing enrichment of GO terms or pathways in old‐specific ligand–receptor interactions. (e). Heatmap showing overlapping significance of aging‐related DEGs between entorhinal cortex and hippocampus

We next used single‐cell regulatory network inference and clustering (SCENIC) to map the gene regulatory networks governing entorhinal cortex aging (Aibar et al., 2017). *FOXN3* is a key regulator of gene expression changes in microglia during entorhinal cortex aging (Figure 3a). GO enrichment analysis indicated that downstream DEGs targeted by *FOXN3* were mainly involved in lipid metabolism and immune system processes (Figure 3b). In addition, we identified a series of TFs (*ZMAT4*, *POU2F1*, *CLK2*, *TCF4*, *MEF2A*, and *SOX6*) that regulate gene expression changes in the neuronal lineage (Figure 3a). GO enrichment analysis indicated that DEGs targeted by major hub TFs in neurons were mainly involved in chemical synaptic transmission and brain development (Figure 3b). These analyses identified the upstream regulons that drive cell‐type‐specific state transitions toward aging.

We next investigated changes in intercellular communication during entorhinal cortex aging. Based on a comprehensive intercellular network of ligand–receptor interactions (Efremova et al., 2020), our results showed that interactions between cell types were globally decreased in the aged group compared with the young group (Figure 3c), indicating weakened intercellular communication in the aged entorhinal cortex. We also found the receptor–ligand pairs specifically present in aged entorhinal cortices were mainly involved in the neuroactive (neuroactive ligand–receptor interactions), pro‐inflammatory (regulation of leukocyte migration), and cell adhesion (positive regulation of cell adhesion) pathways (Figure 3d, Table S5). Thus, these pathways are proposed as mediators of abnormal crosstalk between cell types in the aged entorhinal cortex.

Given the similar functions and frequent information exchange between the entorhinal cortex and hippocampus (Ku, Ku et al., 2021), we next asked whether similar aging mechanisms exist between these two brain regions. By performing comparative analysis of our results and recently published single‐cell hippocampal data from young and old cynomolgus monkeys (Hui Zhang et al., 2021), we found aging‐related DEGs exhibited significant overlapping rate in all cell types between the entorhinal cortex and hippocampus (Figure 3e), suggesting a convergent aging mechanism between these two regions in monkeys.

### 2.4 Transcriptomic reprogramming in aging primate entorhinal cortex is associated with neurological diseases

**FIGURE 4:**
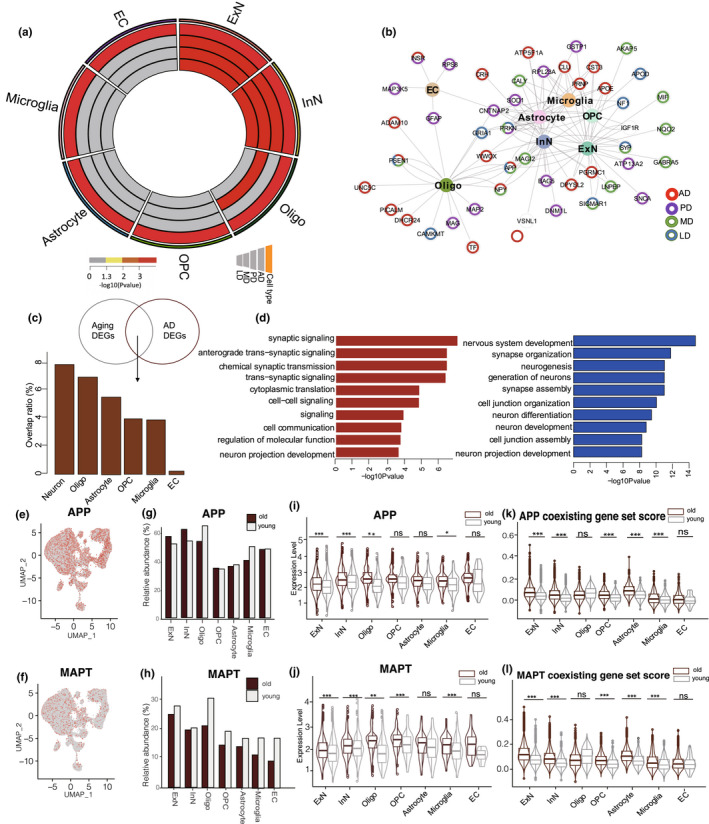
(a). Circos plots showing the significance of difference for of gene set score in AD, PD, MD, and LD across cell types between young and old entorhinal cortex. (b). Network plot showing DEGs associated with aging‐related diseases in different cell types of entorhinal cortex. (c). Genes shared in aging‐DEGs and AD DEGs. Proportion of DEGs shared in aging and AD groups are shown for different cell types. (d). GO term analyses for up(left) and down(right) regulated genes shared by aging‐DEGs and AD DEGs. (e). UMAP plots showing APP‐positive cells in entorhinal cortex. (f). UMAP plots showing MAPT‐positive cells in entorhinal cortex. (g). Percentages of different cell types expressing corresponding APP genes. (h). Percentages of different cell types expressing corresponding MAPT genes. (i). Violin plot showing expression of APP genes across cell types. (j). Violin plot showing expression of MAPT genes across cell types. (k). Violin plot showing gene set score of APP coexisting genes across cell types. (l). Violin plot showing gene set score of MAPT coexisting genes across cell types

Entorhinal cortex aging is a major risk factor for cognitive and memory deficits (Hou et al., 2019). However, how the specific cell types are involved in neurological diseases remains unclear. Based on single‐cell data, we examined cell‐type‐specific expression of genes implicated in AD, Parkinson's disease (PD), and learning and memory disorders (LD, MD) (Aging Atlas, 2021). Results showed that AD‐related genes were significantly up‐regulated in all cell types of the aged entorhinal cortex (Figure 4a), suggesting that cell types in aged entorhinal cortices are widely associated with AD. In addition, genes implicated in PD, LD, and MD were significantly elevated in the aged entorhinal cortex neurons (Figure 4a).

We next constructed a network integrating all cell types, aging‐related DEGs, and risk genes of AD, PD, LD, and MD to identify hub genes in the network (Figure 4b). We identified several disease‐risk genes that were abnormally expressed in specific cell types. Notably, *APOD*, a specific risk gene of LD, showed dysregulated expression in OPCs, while *MAP2*, a specific risk gene for PD, showed dysregulated expression in oligodendrocytes. In addition, several co‐risk genes of diseases were abnormally expressed in multiple cell types. For example, *NPY* (co‐risk gene for AD and MD) showed abnormal expression in both neurons and oligodendrocytes. These results demonstrate the complex network of neurological disease and entorhinal cortex aging at cell level.

Given the key role of entorhinal cortex aging in AD, we performed an integrated analysis of AD‐associated DEGs (AD DEGs) from the human entorhinal cortex (obtained from previous snRNA‐seq data [Grubman et al., 2019]) and aging‐related DEGs from the entorhinal cortex in our study. We identified 166 shared genes between the AD DEGs and aging‐related DEGs (Table S6). These overlapping DEGs were primarily enriched in neurons (Figure 4c), suggesting that the neuronal lineage in the aged entorhinal cortex is more prone to AD. GO analysis showed that the up‐regulated overlapping DEGs were primarily related to synaptic signaling, whereas the down‐regulated overlapping DEGs were mainly associated with neurogenesis (Figure 4d).

### 2.5 The expression of Aβ and NFT increased across multiple cell types in aged entorhinal cortex

The entorhinal cortex is one of the brain regions in which Aβ and NFTs are first detected in old age, both with and without mild cognitive impairment (Thaker et al., 2017). Accumulated Aβ peptides are the main component of senile plaques and are derived from the proteolytic cleavage of the large glycoprotein amyloid precursor protein (APP) (O'Brien & Wong, 2011). Several APP cleavage products are considered as potential contributors to AD, leading to neuronal dysfunction (G.f. Chen et al., 2017). The microtubule‐associated protein tau (MAPT) is responsible for encoding the tau protein, which is strongly implicated in the maintenance of microtubule and axonal transport functions (Strang et al., 2019). Hyperphosphorylated tau protein participates in the formation of NFTs, which characterize many neurodegenerative disorders, termed tauopathies (C.C. Zhang et al., 2016). Here, using our data, we assessed *APP* and *MAPT* expression in the aged entorhinal cortex at cell level. Results showed that *APP* and *MAPT* were widely expressed across all cell types in the entorhinal cortex (Figure 4e, f), but with more *APP*‐ and *MAPT*‐positive cells in the neuronal lineage relative to other cell types (Figure S3). Comparing the proportions of *APP*‐ and *MAPT*‐positive cells between the young and old groups, we found no significant change in the proportion of cells during entorhinal cortex aging (Figure 4g, h), but the expression levels of *APP* and *MAPT* were significantly elevated in most cell types in the aged entorhinal cortex (Figure 4i, j). Furthermore, we evaluated the expression levels of 270 proteins co‐localized with Aβ plaques and 543 proteins co‐localized NFTs based on laser capture microdissection (LCM) and label‐free quantitative (LFQ) proteomic analysis (Drummond et al., 2017; Drummond et al., 2020; Table S7). Results showed that the expression levels of proteins co‐localized with Aβ plaques and NFTs increased significantly in most cell types in the aged entorhinal cortices (Figure 4k, l). Thus, the elevated expression of *APP* and *MAPT*, rather than the number of positive cells expressing *APP* and *MAPT*, was likely the major cause of Aβ deposition and NFT formation in the aged entorhinal cortex.

### 2.6 ExNs subpopulations in aged entorhinal cortex are prone to AD pathology

**FIGURE 5:**
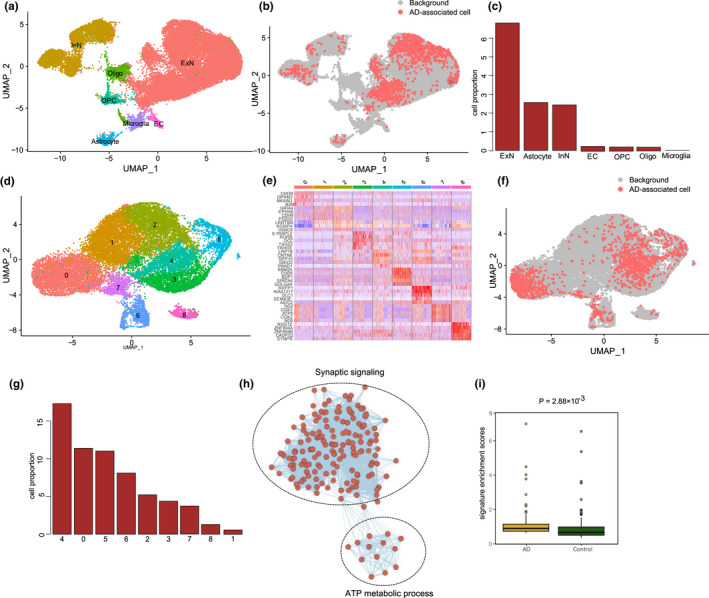
(a). UMAP visualization of cells in aged entorhinal cortex. (b). UMAP visualization of AD‐related cells. Red and gray dots represent cells associated with AD and normal phenotypes, respectively. (c). Bar plot showing constitution of AD‐related cells in different cell types. (d). UMAP visualization of aged ExNs in entorhinal cortex, (e). Heatmap showing expression profiles of subcluster‐specific marker genes in ExNs. (f). UMAP visualization of AD‐related cells in ExNs. Red and gray dots represent cells associated with AD and normal phenotypes, respectively. (g). Bar plot showing constitution of AD‐related cells in different subclusters of ExNs. (h). Enrichmap plot showing biological processes of DEGs between AD‐related and background cells. (i). Box plot shows the enrichment scores of the Scissor_AD ExNs in patients with AD and normal controls

To determine correlations between cell types and AD phenotypes and identify key cell types relevant to AD, we used Single‐Cell Identification of Subpopulations with Bulk Sample Phenotype Correlation (Scissor) (Sun et al., 2021), which can identify cell subpopulations associated with a given phenotype from single‐cell data. Scissor integrates phenotype‐associated bulk expression and single‐cell data by quantifying similarity between each single cell and each bulk sample, then optimizes a regression model on the correlation matrix with the sample phenotype to identify relevant subpopulations (Sun et al., 2021). We applied Scissor to the scRNA‐seq data from the aged entorhinal cortex with bulk transcriptomes from AD and non‐AD entorhinal cortices (Jia et al., 2021) (Figure 5a). Results show that aged entorhinal cortical ExNs were more prone to AD than the other cell types (Figure 5b, c).

Selective vulnerability is a fundamental feature of neurodegenerative diseases, in which different neuronal populations show a gradient of susceptibility to degeneration. ExNs are heterogeneous and include multiple subpopulations with distinct molecular and projection properties (Erwin et al., 2021). Therefore, we applied Scissor, guided by bulk samples with AD, to identify aggressive ExNs cell subpopulations within 31,617 ExNs from the scRNA‐seq dataset of the aged entorhinal cortex (Jia et al., 2021). These cells were separated into 9 clusters (Figure 5d, e), which demonstrated the heterogeneous nature of the ExNs. Scissor identified 1370 cells in the ExNs associated with the patients with AD (defined as Scissor_AD ExNs thereafter; Figure 5f). The Scissor_AD ExNs were mainly from clusters 4, 0, and 5 (Figure 5g). To understand the underlying transcriptional patterns of Scissor_AD ExNs, we compared the gene expressions of those cells with all other cells. As a result, 196 up‐regulated genes and 16 down‐regulated genes were differentially expressed in Scissor_AD ExNs over all other cells, respectively (Table S8). Notably, functional enrichment analysis also confirmed that the synaptic signaling and adenosine triphosphate (ATP) metabolic processes were activated in Scissor_AD ExNs (Figure 5h). To further demonstrate the phenotypic associations of the cell subpopulations identified by Scissor, we constructed molecular signatures based on the DEGs in Scissor‐identified cell subpopulations and used independent AD datasets to evaluate the functions of these signatures (Jia et al., 2021). As a result, the enrichment scores of the corresponding molecular signatures in A Scissor_AD ExNs were significantly higher in patients with AD than in normal controls (Figure 5i). Thus, this Scissor_AD ExNs subpopulation could play a vital role in AD progress.

Taken together, Scissor analysis identified ExNs subpopulations that are most highly associated with AD, which could contribute to comprehending the underlying pathogenesis of AD and might facilitate disease diagnosis and therapy.

## 3 DISCUSSION

The entorhinal cortex plays a key role in cognition and memory and is an information exchange center for multiple brain areas (Gerlei et al., 2021). Abnormal entorhinal cortex function is implicated in multiple neurodegenerative diseases (Reagh et al., 2018). However, this brain region has received less attention than other regions such as the hippocampus and prefrontal cortex. In the current study, we used cynomolgus monkeys to construct a single‐cell map of the entorhinal cortex and identify age‐associated transcriptional changes. Our findings suggested widespread transcriptional changes across multiple cell types during entorhinal cortex aging, thus highlighting potential therapeutic targets for aging‐related neurodegenerative disorders.

Our results showed that the synapse signaling‐related pathway was widely down‐regulated across cell types in the aged entorhinal cortex. Cell communication characterized by ligand–receptor interactions was also globally decreased during entorhinal cortex aging, suggesting an abnormal cell microenvironment in the aged entorhinal cortex. Inactive synapse signaling and weak cell communication in the aged entorhinal cortex would likely delay information transfer across cell types and contribute to eventual cognitive decline and memory loss.

We also observed acute changes in the neuronal lineage during entorhinal cortex aging. Specifically, we found the highest number of aging‐related DEGs was observed in the neuronal lineage; genes associated with aging, AD, PD, MD, and LD were significantly more active in the neuronal lineages; and the overlap in aging‐DEGs and AD DEGs was most notable in the neuronal lineage. Therefore, our results confirmed that the neuronal lineage was more vulnerable to aging in the entorhinal cortex and more susceptible to neurological disease.

We systematically explored the association between entorhinal cortex aging and AD (Reagh et al., 2018). Integrative analysis reveals a huge overlap between aging DEGs and AD DEGs across cell types. A hallmark of AD pathology is the accumulation of Aβ and phosphorylated tau (Iwata et al., 2014). In our study, *APP* and *MAPT* gene expression levels, as well as their coexistence, were significantly increased in most cell types in the aged entorhinal cortex, which is likely an important inducement of early AD. Furthermore, based on integration of bulk transcriptome data of AD, we identified ExNs subpopulations that are involved in synaptic signaling and ATP metabolic pathways are most highly associated with AD, which provide potential therapies for the diagnosis and treatment of AD.

## 4 EXPERIMENTAL PROCEDURES

### 4.1 Nuclear isolation from NHP entorhinal cortex

The use of cynomolgus monkey's entorhinal cortex in this study was from the Jing J Kang biotechnology company (Approval number: SCXK 2018–0002). The source animals of these tissues were confirmed to have no disease history and natural death. Entorhinal cortex were stored in −80°C and washed in pre‐cooled PBSE (PBS buffer containing 2 mM EGTA) before the start of the experiment. Nucleus isolation was carried out using GEXSCOPE® Nucleus Separation Solution (Singleron Biotechnologies, Nanjing, China) refer to the manufacturer's product manual. Isolated nuclei were resuspended in PBSE to 106 nuclei per 400 μl, filtered through a 40 μm cell strainer, and counted with Trypan blue. Nuclei enriched in PBSE were stained with DAPI (1:1000) (Thermo Fisher Scientific, D1306). Nuclei were defined as DAPI‐positive singlets. Nuclear isolation was carried out using GEXSCOPE® Nucleus Separation Solution (Singleron Biotechnologies, Nanjing, China) per the manufacturer's product manual. Isolated nuclei were resuspended in PBSE to 10^6^ nuclei/400 μl, filtered through a 40‐μm cell strainer, and counted with Trypan blue. Nuclei enriched in PBSE were stained with DAPI (1:1000; Thermo Fisher Scientific, D1306). Nuclei were defined as DAPI‐positive singlets.

### 4.2 Single‐nucleus RNA‐sequencing library preparation

The concentration of the single‐nucleus suspension was adjusted to 3 ~ 4 × 10^5^ nuclei/mL in PBS and then loaded onto a microfluidic chip (GEXSCOPE® Single Nucleus RNA‐seq Kit, Singleron Biotechnologies). The snRNA‐seq libraries were constructed according to the manufacturer's instructions (Singleron Biotechnologies). The resulting snRNA‐seq libraries were sequenced on an Illumina HiSeq X10 instrument to a sequencing depth of at least 50,000 reads per cell with 150‐bp paired‐end (PE150) reads.

### 4.3 Generation of single‐cell gene expression matrices

Raw reads were processed to generate gene expression matrices with scopetools (https://anaconda.org/singleronbio/scopetools). First, reads without polyT tails were filtered; then, cell barcodes and unique molecular identifiers (UMIs) were extracted. Adapters and polyA tails were trimmed before aligning reads to the pre‐mRNA reference (Ensemble, Macaca_fascicularis_6.0). Second, reads with the same cell barcode, UMI, and gene were grouped together to count the number of UMIs per gene per cell. Cell number was then determined according to the “knee” method, a standard single‐cell RNA‐seq quality control approach used to determine the threshold at which cells are considered valid for experimental analysis. High‐quality barcodes are located to the left of the inflection (“knee”) point and retained for further analysis, while low‐quality barcodes (i.e., relatively low numbers of reads) are located to the right and excluded from further analysis.

### 4.4 Quality control, cell‐type clustering, and major cell‐type identification

We removed cells that had either <200 or >4000 expressed genes. Low‐quality/dying cells often exhibit extensive mitochondrial contamination. Therefore, we applied the “PercentageFeatureSet” function in the Seurat R package (version = 4.0) to calculate the percentage of counts originating from mitochondrial genes (Figure S4), with cells showing a mitochondrial ratio greater than 1.5% discarded. Finally, 76,839 cells were obtained for downstream analysis. Harmony was used as the batch effect removal method to reduce heterogeneity among cells of an individual.

We used Seurat v4.0 to normalize expression matrices using the NormalizeData and ScaleData functions. The FindVariable function was then applied to select the top 200 variable genes and perform principal component analysis (PCA). The first 10 principal components (PCs) and resolution 1.3 were used with the FindClusters function to generate 32 cell clusters. To assign one of the seven major cell types to each cluster, we scored each cluster by the normalized expression levels of the following canonical markers: astrocytes (*AQP4*, *ADGRV1*, *GPC5*, *RYR3*), ECs (*CLDN5*, *ABCB1*, *EBF1*), ExNs (*CAMK2A*, *CBLN2*, *LDB2*), InNs (*GAD1*, *LHFPL3*, *PCDH15*), microglia (*C3*, *LRMDA*, *DOCK8*), oligodendrocytes (*MBP*, *PLP1*, *ST18*), and OPCs (*PDGFRA*, *MEGF11*, *OLIG1*). The clusters assigned to the same cell type were grouped together for the following analyses. The results were manually examined to ensure the correctness of the results and visualized using UMAP. Marker genes for each cell type were identified using the “FindAllMarkers” function with an adjusted *p* < 0.05 and |logFC| > 1 cutoff.

### 4.5 Age‐related DEG analysis

DEGs for every cell type between young and old samples were identified with the “FindMarkers” function in Seurat *R* package using the Wilcoxon test (adjusted *p* < 0.05 and |logFC| > 0.25 threshold).

### 4.6 Identifying statistically significant differences in cell proportions

we used the method reported by Smillie et al. (Smillie et al., 2019), to identify changes in cell proportions between young and aged NHP entorhinal cortex. We applied Dirichlet‐multinomial regression model, which tests for differences in cell composition between young and aged NHP entorhinal cortex. This regression model and its associated p values were calculated using the “DirichReg” function in the DirichletReg R package.

### 4.7 Transcriptional regulatory network analysis

Transcription factor regulatory network analysis was performed using the pySCENIC workflow (v1.1.2.2) with default parameters. We downloaded hg19 TFs using RcisTarget (v1.6.0) as a reference. Gene regulatory networks were inferred with GENIE3 (v1.6.0).

### 4.8 GO term analysis

The clusterProfiler R package and Metascape were used to perform GO term analysis (http://metascape.org/gp/index.html) (v3.5) (Zhou et al., 2019). Results were visualized using the ggplot2 R package (https://ggplot2.tidyverse.org/) (v3.2.1).

### 4.9 Cell–cell communication analysis

Cell–cell communication analysis was performed using Cell‐PhoneDB (v1.1.0) (Efremova et al., 2020). Only receptors and ligands expressed in more than 10% of cells of any type from either young or old samples were further evaluated. Only those with *p* < 0.01 were used for cell–cell communication prediction between any two cell types.

### 4.10 Gene set score analysis

Gene sets related to aging‐related diseases were previously reported (Aging Atlas, 2021). Gene set scores were acquired by analyzing the transcriptome of each input cell against the aforementioned gene sets using the Seurat function “AddModuleScore.” Changes in scores between young and old samples were analyzed using the ggpubr R package via the Wilcoxon test (https://github.com/kassambara/ggpubr) (v0.2.4).

### 4.11 Scissor analysis for each cell type

Three data sources are used for Scissor input: that is, single‐cell expression matrix, bulk expression matrix, and phenotype of interest. Given the above inputs, we used Scissor to select the phenotype‐associated cell subpopulations, which were fit by a binomial regression model (family = “binomial”). We set the parameter alpha (α) = 0.01 to choose AD‐related cells.

## AUTHOR CONTRIBUTIONS

Yuming Xu leads the project. Yuming Xu and Ming‐Li Li and Shi‐Hao Wu designed and conceived the study. Ming‐Li Li, Bo Song, Jing Yang and Li‐Yuan Fan. Jing‐Hua Yang drafted the manuscript. Ming‐Li Li performed data analysis. Shi‐Hao Wu, Yang and Yun‐Chao Wang performed the functional experiments.

## CONFLICT OF INTEREST

The authors declare no competing financial interest.

## Supporting information


**Figure S1** (a) Immunofluorescence staining of Aβ (4G8) accumulation in the Entorhinal cortex from young and old monkeys. Scale bars, 100 μm.** *p* < 0.01. (b) Nissl staining of entorhinal cortex in young and old monkeys. Scale bars, 50 μm (zoomed‐in image). ** *p* < 0.01.
**Figure S2**. Cell proportion changes between young and aged NHP entorhinal cortex. Shown are significant changes in cell frequency (y axis) for aged samples (red) relative to young (blue) (Dirichlet‐multinomial regression, ns = not significant); error bars: SEM.
**Figure S3**. Percentages of different cell types expressing the APP‐positive gene.
**Figure S4**. Percentage of mitochondrial genes detected in each sample.

**Table S1** The sample information of cynomolgus monkey in this study
**Table S2**: cell type‐specific markers in entorhinal cortex
**Table S3**: upstream regulators that drive cell differentiation in entorhinal cortex
**Table S4**: Differently expressed genes between young and old group across cell types
**Table S5**: old‐specific ligand–receptor interactions
**Table S6**: the overlapping gene list of AD DEGs and aging DEGs
**Table S7**: list of APP coexist gene and MAPT coexist gene
**Table S8**: DEGs between Scissor_AD ExNs and other cells

## Data Availability

All the sequencing data are deposited in Genome Sequence Archive (GSA) (https://bigd.big.ac.cn/gsa/) with the accession number of CRA006617.
