# Supplementary material for: Single‐cell analysis reveals transcriptomic reprogramming in aging primate entorhinal cortex and the relevance with Alzheimer's disease
Source: Aging Cell. 2022 Sep 27;21(11):e13723. doi: 10.1111/acel.13723 (PMC9649611; doi:10.1111/acel.13723)
Supplement: Supplementary file 1 — Figure S1 (a) Immunofluorescence staining of Aβ (4G8) accumulation in the Entorhinal cortex from young and old monkeys. Scale bars, 100 μm.** p < 0.01. (b) Nissl staining of entorhinal cortex in young and old monkeys. Scale bars, 50 μm (zoomed‐in image). ** p < 0.01. Figure S2. Cell proportion changes between young and aged NHP entorhinal cortex. Shown are significant changes in cell frequency (y axis) for aged samples (red) relative to young (blue) (Dirichlet‐multinomial regression, ns = not significant); error bars: SEM. Figure S3. Percentages of different cell types expressing the APP‐positive gene. Figure S4. Percentage of mitochondrial genes detected in each sample. [file ACEL-21-e13723-s001.docx]

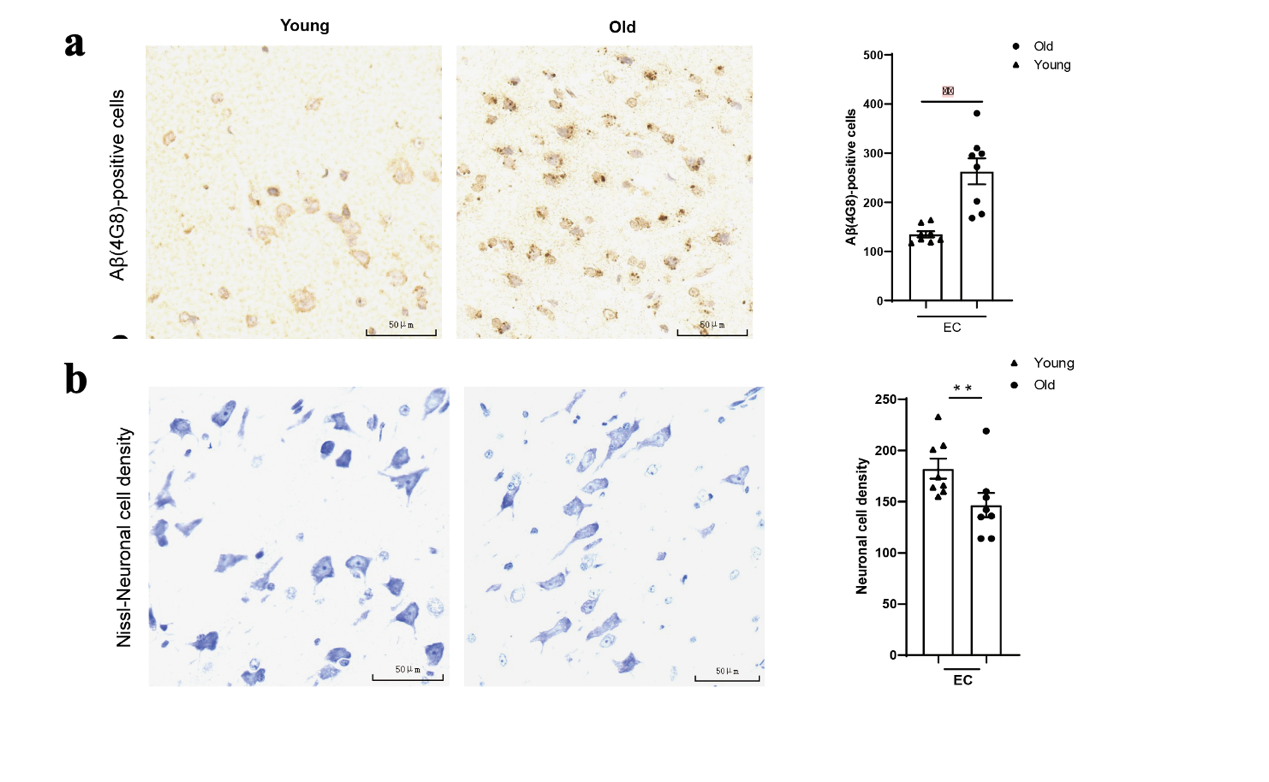


Figure S1. a. Immunofluorescence staining of Aβ (4G8) accumulation in the Entorhinal cortex from young and old monkeys. Scale bars, 100 μm.** P < 0.01.b. Nissl staining of entorhinal cortex in young and old monkeys. Scale bars, 50 μm (zoomed-in image). ** *P* < 0.01.

**
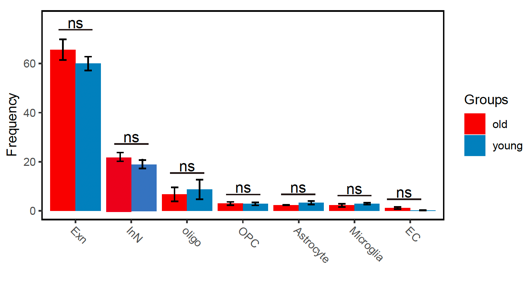
**

Figure S2. Cell proportion changes between young and aged NHP entorhinal cortex. Shown are significant changes in cell frequency (y axis) for aged samples (red) relative to young (blue) (Dirichlet-multinomial regression, ns=not significant); error bars: SEM.


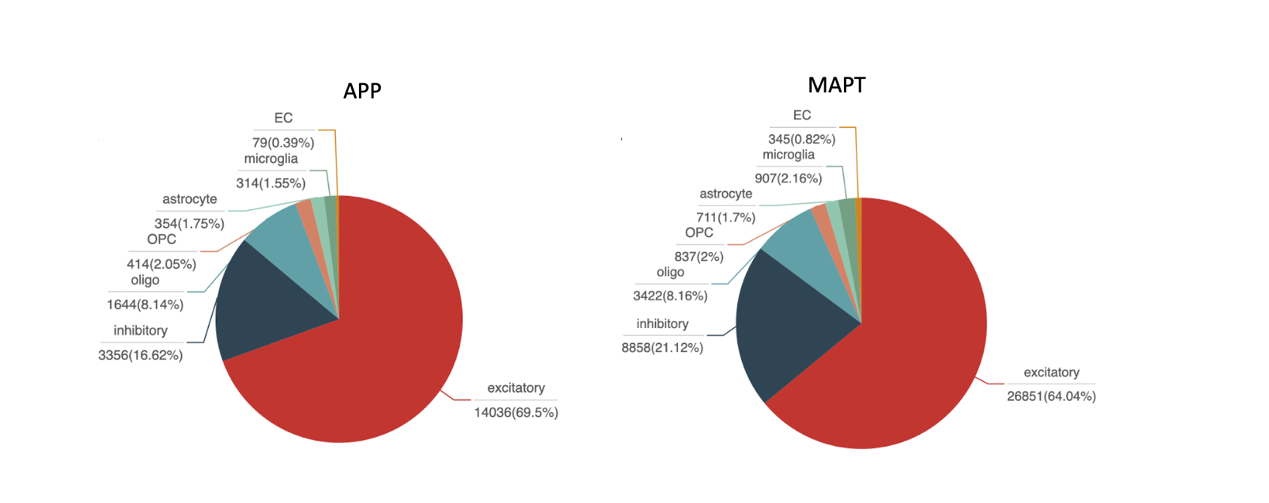


Figure S3. Percentages of different cell types expressing the APP positive gene.


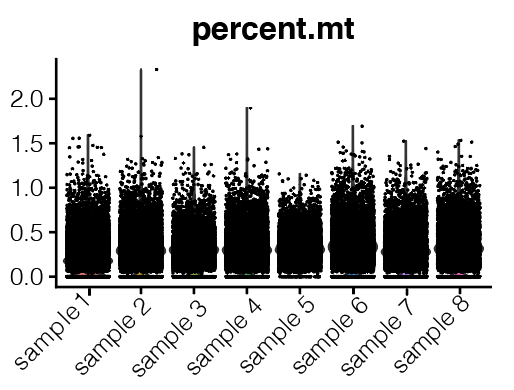


Figure S4. Percentage of mitochondrial genes detected in each sample.
